# Primary care physician beliefs about insulin initiation in patients with type 2 diabetes

**DOI:** 10.1111/j.1742-1241.2008.01742.x

**Published:** 2008-06

**Authors:** R P Hayes, J T Fitzgerald, S J Jacober

**Affiliations:** 1 Global Health Outcomes, Eli Lilly and Company Indianapolis, IN, USA; 2 Department of Medical Education, University of Michigan Ann Arbor, MI, USA; 3 Lilly Research Laboratories, Eli Lilly and Company Indianapolis, IN, USA

## Abstract

**Background:**

Insulin is the most effective drug available to achieve glycaemic goals in patients with type 2 diabetes. Yet, there is reluctance among physicians, specifically primary care physicians (PCPs) in the USA, to initiate insulin therapy in these patients.

**Aims:**

To describe PCPs’ attitudes about the initiation of insulin in patients with type 2 diabetes and identify areas in which there is a clear lack of consensus.

**Methods:**

Primary care physicians practicing in the USA, seeing 10 or more patients with type 2 diabetes per week, and having > 3 years of clinical practice were surveyed via an internet site. The survey was developed through literature review, qualitative study and expert panel.

**Results:**

Primary care physicians (*n* = 505, mean age = 46 years, 81% male, 62% with > 10 years practice; 52% internal medicine) showed greatest consensus on attitudes regarding risk/benefits of insulin therapy, positive experiences of patients on insulin and patient fears or concerns about initiating insulin. Clear lack of consensus was seen in attitudes about the metabolic effects of insulin, need for insulin therapy, adequacy of self-monitoring blood glucose, time needed for training and potential for hypoglycaemia in elderly patients.

**Conclusions:**

The beliefs of some PCPs are inconsistent with their diabetes treatment goals (HbA1c ≤ 7%). Continuing medical education programmes that focus on increasing primary care physician knowledge about the progression of diabetes, the physiological effects of insulin, and tools for successfully initiating insulin in patients with type 2 diabetes are needed.

**Disclosures:**

Drs Hayes and Jacober are employees and stockholders of Eli Lilly and Company. Dr Fitzgerald is a consultant to Eli Lilly and Company.

**What's known**

Insulin is the most effective drug available to achieve glycaemic goals in patients with type 2 diabetes, yet there is reluctance among many physicians to initiate insulin therapy in these patients. Diabetes specialists tend to be more aggressive than primary care physicians (PCPs) with insulin initiation in patients with type 2 diabetes, and US physicians are more disposed to delay insulin than physicians in other countries.

**What's new**

This article confirms that US PCPs lack consensus on some beliefs about insulin initiation. Consensus was seen regarding insulin risk/benefits, positive patient experiences of insulin and patient fears about initiating insulin. No consensus was seen regarding insulin's metabolic effects, need for insulin, adequacy of self-monitoring blood glucose, time needed for training and potential for hypoglycaemia in elderly patients. Some PCPs have beliefs inconsistent with their diabetes treatment goals (HbA1c ≤ 7%).

## Introduction

Patients with type 2 diabetes are frequently maintained in poor glycaemic control for prolonged periods, increasing the risk of serious complications (1). Insulin is the most effective therapy to achieve glycaemic goals in these patients (2), yet there is reluctance among patients and physicians to initiate insulin (3).

Contributors to patient reluctance to initiate insulin include concerns about side effects, desire to avoid injections, feelings of personal failure and skepticism about insulin's effectiveness (3–5). Less is known about the factors that contribute to physician reluctance to initiate insulin in patients with type 2 diabetes. Riddle (6) observed patterns of insulin usage in the USA and found little consensus among medical practitioners regarding when insulin therapy should be initiated. He proposed that some providers were reluctant to prescribe insulin to patients with type 2 diabetes because of both theoretical concerns (hypoglycaemia, weight gain and the belief that insulin has negative metabolic effects) and practical concerns (patient anxiety about insulin, patient cognitive abilities and the complexity of training patients to administer insulin). Riddle noted that diabetes specialists tended to be more aggressive than primary care physicians (PCPs) with insulin initiation in patients with type 2 diabetes.

The Diabetes Attitude Wishes and Needs study (3), a large multinational survey of physicians and patients, indicated that US physicians were significantly more disposed to delay insulin therapy than physicians in most other countries. The results also indicated that diabetes specialists are less inclined than PCPs to delay insulin initiation. A recent US survey of diabetes specialists and academic generalists showed that specialists reported no major barriers to initiating insulin treatment in patients with type 2 diabetes, but the majority of academic generalists indicated several patient-derived barriers (e.g. patients’ fear of insulin) (7).

While these studies provide some insight into US physicians’ attitudes about initiating insulin in patients with type 2 diabetes, they did not include PCPs, who care for the majority of patients with type 2 diabetes in the USA (8).

This study aimed to describe the attitudes of US PCPs regarding initiating insulin in patients with type 2 diabetes and addresses these research questions:

- Is there consensus among PCPs on some beliefs about insulin initiation; if so, which ones?
- Is there lack of consensus among PCPs on some beliefs about insulin initiation; if so, which ones?
- Are there associations between PCP characteristics (such as age, years of practice, etc.) and beliefs about insulin therapy?

## Methods

### Study participants

Study participants were sampled from the physician panel of Harris Interactive, a large market research firm. The panel consists of more than 40,000 US physicians, is representative of the general US physician population and includes more than 40 medical specialties and several subspecialties. Physician names are continuously updated and authenticated against the American Medical Association (AMA) master file. Panel membership is voluntary; physicians may unsubscribe at anytime. To qualify for the study, physicians were required to have > 3 years of clinical practice experience and to treat > 10 patients with type 2 diabetes per week.

### Physician survey development

The physician survey included a demographic assessment, a question about glycaemic goals for three patient age groups, and 30 belief items beginning with ‘I believe…’. For the belief items, respondents were asked to indicate on a five-point Likert-type scale ranging from one, ‘strongly disagree’, to five, ‘strongly agree’, the extent to which they agreed with the statements presented.

Item development for the survey was based on a review of the literature on physician barriers to initiating insulin in patients with type 2 diabetes (3,6,9), a pilot study of web-based case studies (10), results of an online asynchronous focus group (in which participants log on at their convenience) of 15 PCPs (unpublished data, 2005), and the input of an expert panel [two PCPs and two authors of the Diabetes Attitude Scale (RMA, JTF)] (11). Items were categorised *a priori* as beliefs about insulin as an injection, metabolic effect of insulin, risk/benefit of insulin, perceived concerns about insulin therapy of patients on oral therapy, perceived experiences of patients on insulin therapy, appropriate timing for insulin therapy initiation, and the training and resources needed for insulin therapy initiation. Some items were worded with the expectation that most PCPs would agree with the statement, others with the expectation that most PCPs would disagree with the statement.

### Survey recruitment and administration

Harris Interactive fielded the online survey from 19 December 2005 to 21 December 2005. Email invitations were sent to 2552 physicians board certified in Family Practice, General Practice or Internal Medicine. Recipients were offered a $60 honorarium or the option to donate the honorarium to a charity of the recipient's choice upon qualifying for and completing the survey before the quota of responses (505 responses, determined by the funding available for the honoraria with an approximately equal representation of internal medicine and family practice physicians) was filled. The survey was accessible to invited physicians until the quota was filled. Of the 982 physicians (39% of the recipients) who responded to the invitation, 505 (51%) qualified for and completed the survey, 70 (7%) did not qualify, and 407 (41%) qualified after the quota had been met and did not complete the survey.

### Statistical analysis

Frequency distributions were calculated for all survey items. To determine beliefs about which PCPs lacked consensus, the ‘strongly agree’ responses were combined with the ‘agree’ responses and the ‘strongly disagree’ responses were combined with the ‘disagree’ responses. Items with 50% or more of responses falling into either the ‘agree’ or the ‘disagree’ category were considered beliefs about which there was consensus. Items for which neither the ‘agree’ nor the ‘disagree’ category contained 50% or more of the responses were considered beliefs about which there was no consensus and which may represent areas of confusion.

To identify associations between selected PCP characteristics and item responses, a one-way analysis of variance (ANOVA) was performed for each of the 30 items using the item as a dependent variable and PCP characteristics as independent variables. Scheffe *post hoc* tests were used to determine significant differences between independent variable groups. Because a large number of statistical tests were performed, alpha was set at < 0.01.

## Results

### PCP characteristics

The average age of respondents (*n* = 505) was approximately 46 years; 81% were male and 62% had been in practice for > 10 years. Fifty-two per cent of the PCPs were board certified in internal medicine, and 78% reported seeing an average of 10–59 patients a week (Table 1).

**Table 1:** Primary care physician characteristics glycaemic control treatment goals for different age groups

Physician characteristics	Total (*n* = 505)
Mean age, years (SD)	45.6 (8.7)
Male gender, *n* (%)	407 (81)
**Years in practice, n (%)**	
3–5 years	55 (11)
6–10 years	139 (27)
11–15 years	84 (17)
16–30 years	215 (43)
More than 30 years	12 (2)
**Primary care board certification, *n* (%)**	
Internal medicine	262 (48)
Family practice	231 (50)
General practice	12 (2)
**Number of patients with type 2 diabetes seen in an average week, *n* (%)**	
10–25	172 (34)
26–59	221 (44)
60–99	60 (12)
100+	52 (10)
**Treatment goal (HbA1c ≤ 7%), *n* (%)**	
Younger than 50 years of age	498 (99)
51–69 years of age	474 (94)
More than 70 years of age	407 (81)

Nearly, all PCPs indicated that their HbA1c goal was ≤ 7% for patients with type 2 diabetes < 50 years old (99%) or 50–70 years old (94%), and 81% indicated that their HbA1c goal was ≤ 7% for patients > 70 years old.

### Research question 1: shared beliefs

Table 2 presents the response distributions of belief items for which > 50% of responses fell into the ‘agree’ or ‘disagree’ category. For the first 13 items listed, ≥ 66% of PCPs agreed with the statement, indicating shared beliefs about the barrier that injection poses to patients’ acceptance of insulin (items 1, 3) and physician prescribing of insulin (item 6), the importance of education to insulin initiation (item 2), the benefits of insulin outweighing risks of hypoglycaemia (item 4) and weight gain (item 5), the benefit to patients of receiving insulin prior to the development of complications (item 10), patient fears prior to starting insulin (item 8), the physical improvements in (item 9) and coping ability of (items 7, 12) patients once they are on insulin, the reluctance of patients on oral therapy to accept an insulin prescription (item 11), and the initiation of insulin as one of the most difficult aspects of managing patients with type 2 diabetes (item 13).

**Table 2:** Frequency distributions for items in which 50% or more primary care physicians (*n* = 505) ‘agreed’ or ‘disagreed’ with the statement in the order of descending agreement

	Response		
	
Items	Disagree to strongly disagree (%)	Neutral (%)	Agree to strongly agree (%)
**I believe…**			
1. …more of my patients would be willing to initiate insulin therapy if it were not administered by injection	2	5	93
2. …for most of my patients, education is the key to the initiation of insulin	2	5	93
3. …for most of my patients, the injection route of administration is the greatest barrier to their acceptance of insulin therapy	3	8	89
4. …for most of my patients, the benefits of insulin therapy outweigh the risks of hypoglycaemia	4	9	88
5. …for most of my patients, the benefits of insulin therapy outweigh the risks of weight gain	2	10	88
6. …primary care physicians might prescribe insulin more frequently if the route of administration did not involve injections	7	10	83
7. …most of my patients using insulin are able to manage the demands of insulin therapy	4	15	82
8. …most of my patients on oral diabetes therapy are afraid of insulin therapy	6	14	80
9. …most of my patients using insulin feel much better physically once they become accustomed to using insulin therapy	4	20	76
10. …most patients would benefit from receiving insulin therapy prior to the development of diabetes complications	7	18	75
11. …most of my patients on oral diabetes therapy would be reluctant to accept a prescription for insulin	11	18	72
12. …most of my patients find the demands of insulin therapy to be less than they expected	12	19	69
13. …the initiation of insulin is one of the most difficult aspects of managing my patients with type 2 diabetes	19	15	66
14. …most of my patients using insulin take their insulin as prescribed (i.e. are adherent)	13	24	63
15. …most of my patients on oral diabetes therapy would regard the initiation of insulin as a personal failure	21	26	53
16. …most of my patients using insulin are satisfied with their diabetes therapy	19	28	53
17. …training in the proper administration and usage of insulin is too complicated for most patients	58	22	20
18. …the follow-up needed for most of my patients on insulin is too resource-intensive for my staff	53	22	25
19. …the risk of weight gain associated with insulin therapy makes me reluctant to prescribe it for most of my patients with BMI ≥ 35	50	23	27
20. …for most of my patients, the fear of side effects (hypoglycaemia and/or weight gain) is the greatest barrier to their acceptance of insulin therapy	50	24	26

BMI, body mass index.

At least 50% of PCPs agreed that most of their patients on insulin are using their insulin as prescribed (item 14) and are satisfied with their diabetes therapy (item 16). More than half also agreed that most of their patients on oral therapy would regard insulin initiation as a personal failure (item 15).

The majority of PCPs disagreed that the follow-up needed for most patients was too resource-intensive for their staff (item 17) or that training in insulin administration is too complicated for most patients (item 18). The majority also disagreed that the risk of weight gain made them reluctant to prescribe insulin to patients with body mass index (BMI) ≥ 35 (item 19) and that the fear of side effects is the greatest barrier to patients’ acceptance of insulin therapy (item 20).

### Research question 2: beliefs about which PCPs lack consensus

Table 3 presents response distributions for the belief items in which < 50% of responses fell into the ‘agree’ or ‘disagree’ category. Forty-four per cent of PCPs agreed that the risk of hypoglycaemia would make them reluctant to prescribe insulin for most patients ≥ 85 years old (item 1), 44% disagreed that most patients on oral diabetes therapy would be less adherent with insulin therapy, and 45% disagreed that they should wait until patients on oral therapy have a beta cell inadequacy to prescribe insulin (items 9, 10).

**Table 3:** Frequency distributions for items in which < 50% of primary care physicians ‘agreed’ or ‘disagreed’ with the statement in the order of descending agreement

	Response		
	
Items	Disagree to strongly disagree (%)	Neutral (%)	Agree to strongly agree (%)
**I believe…**			
1. …the risk of hypoglycaemia from insulin therapy makes me reluctant to prescribe it for most of my patients ≥ 85 years of age	32	24	44
2. …most of my patients using insulin self-monitor their blood glucose with sufficient frequency	40	18	43
3. …training most of my patients in the proper administration and usage of insulin is too time-consuming for my staff	38	21	40
4. …most patients would not need to go on insulin if they would follow their physicians’ recommendations	36	24	40
5. …most patients will eventually need to go on insulin regardless of how well they adhere to their treatment regimen	41	20	39
6. …insulin therapy has a beneficial effect on insulin resistance	30	32	39
7. …increased levels of plasma insulin will increase the risk of a cardiovascular event	32	33	35
8. …increasing insulin levels in obese patients will cause more insulin resistance	31	38	31
9. …most patients do not need a prescription of insulin until they have a beta cell inadequacy	44	25	31
10. …most of my patients on oral diabetes therapy would be less adherent with insulin therapy	45	29	27

Response distributions for items 2 through 5 in Table 3 show bimodal distributions (< 25% of the responses were ‘neutral’, and remaining responses were nearly equally divided between ‘agree’ and ‘disagree’). The results indicate that PCPs lack consensus that: most patients using insulin self-monitor their blood glucose with sufficient frequency, patients can avoid insulin therapy by following their physicians’ recommendations, patients will need insulin therapy regardless of treatment adherence, and the time needed for training patients to use insulin is too much for their staff.

Item response distributions for items 6 through 8 in Table 3 show unimodal distributions (responses are distributed approximately equally between ‘agree’, ‘neutral’ and ‘disagree’), suggesting that there is confusion among PCPs about the metabolic effect of insulin.

### Research question 3: associations between beliefs and PCP characteristics

#### Gender

One significant difference in beliefs by gender was identified: Women agreed significantly (p < 0.01) more strongly than men (3.58 vs. 3.30) that ‘…most of my patients on oral therapy would regard the initiation of insulin as a personal failure’.

#### Years of practice

Few PCPs in the study had practiced > 30 years, so data from those PCPs were combined with data from PCPs who had practiced 16–30 years for the one-way ANOVA procedures. *Post hoc* analyses showed significant (p < 0.01) differences between categories of years of practice for seven belief items (Table 4). In general, PCPs with > 15 years experience agreed more strongly than those with ≤ 15 years experience that patients on insulin were able to cope with and adhere to insulin therapy (p = 0.004, p < 0.001 respectively). In addition, the more experienced group disagreed more strongly than the less experienced group that training and follow-up for patients on insulin were too resource-intensive or complex. PCPs who had practiced 3–5 years disagreed more strongly than one or more of the groups that had practiced > 5 years that insulin has negative metabolic effects.

**Table 4:** Belief items with significant (p < 0.01) one-way analysis procedures and at least one significant *post hoc* test by years of practice

	Years of practice, mean score (SD)				
		
Belief items	3–5 (*n* = 55)	6–10 (*n* = 139)	11–15 (*n* = 84)	> 15 (*n* = 227)	p-value (ANOVA)
**I believe that…**					
…most of my patients using insulin feel much better physically once they become accustomed to using insulin therapy	4.0* (0.6)	3.6 (0.7)	3.8 (0.6)	3.9 (0.7)	0.002
…the follow-up needed for most of my patients on insulin is too resource-intensive for my staff	2.8 (1.0)	2.9 (1.0)	2.8 (1.1)	2.5† (0.9)	0.001
…training in the proper administration and usage of insulin is too complicated for most patients	2.8 (0.9)	2.8 (0.9)	2.6 (1.0)	2.4† (0.8)	< 0.001
…most of my patients using insulin are able to manage the demands of insulin therapy	3.8 (0.7)	3.7 (0.6)	3.8 (0.5)	3.9† (0.5)	0.004
…most of my patients using insulin take their insulin as prescribed (i.e. are adherent)	3.3 (0.9)	3.5 (0.7)	3.3 (0.8)	3.7‡,§ (0.7)	< 0.001
…increased levels of plasma insulin will increase the risk of a cardiovascular event	2.6* (1.0)	3.2 (0.9)	3.2 (0.9)	3.1 (0.9)	0.002
… increasing insulin levels in obese patients will cause more insulin resistance	2.6*,¶ (1.0)	3.1 (0.8)	3.2 (0.9)	3.0 (0.9)	< 0.001

* 3–5 years are significantly different from 6 to 10 years.

† > 15 years are significantly different from 6 to 10 years.

‡ > 15 years are significantly different from 3 to 5 years.

§ > 15 years are significantly different from 11 to 15 years.

¶ 3–5 years are significantly different from 11 to 15 years.

#### Primary care certification

For the one-way ANOVA procedures, data from general practitioners were combined with data from family practitioners because there were few general practitioners in the study. One significant (p < 0.01) difference in beliefs was found between internists and family/general practitioners: family/general practitioners agreed more strongly that ‘…most patients would not need to go on insulin if they would follow their physicians’ recommendations’ (3.21 vs. 2.97).

#### Average number of patients with type 2 diabetes seen per week

*Post hoc* analyses showed significant (p < 0.01) differences between categories of number of patients with type 2 diabetes seen per week for five belief items (Table 5). Mean scores of PCPs who saw ≥ 100 such patients per week were significantly different from one or more of the categories of PCPs who saw fewer such patients for all five items. PCPs seeing ≥ 100 such patients agreed more strongly that patient fear of side effects was the greatest barrier to acceptance of insulin, the risk of weight gain made them reluctant to use insulin in patients with BMI ≥ 35, follow-up for patients on insulin is too resource-intensive, training in proper use of insulin is too complicated for most patients, and patients on oral therapy would be less adherent with insulin therapy.

**Table 5:** Belief items with significant (p < 0.01) one-way analysis procedures and at least one significant *post hoc* test by average number of patients with type 2 diabetes seen per week

	Average number of patients with type 2 diabetes seen per week, mean score (SD)				
		
	10–25 *(n* = 172)	26–59 *(n* = 221)	60–99 *(n* = 60)	≥ 100 *(n* = 52)	p-value (ANOVA)
**I believe…**					
…for most of my patients, the fear of side effects (hypoglycaemia and/or weight gain) is the greatest barrier to their acceptance of insulin therapy	2.6 (0.9)	2.8 (1.0)	2.9 (1.1)	3.3*,† (1.0)	< 0.001
…the risk of weight gain associated with insulin therapy makes me reluctant to prescribe it for most of my patients with BMI ≥ 35	2.7 (1.0)	2.6 (1.0)	2.9 (1.0)	3.2† (1.0)	0.001
…the follow-up needed for most of my patients on insulin is too resource-intensive for my staff	2.6 (0.9)	2.6 (1.0)	2.8 (1.0)	3.2† (1.1)	0.001
…training in the proper administration and usage of insulin is too complicated for most patients	2.5 (0.8)	2.5 (0.9)	2.8 (1.0)	3.0*,† (1.0)	< 0.001
…most of my patients on oral diabetes therapy would be less adherent with insulin therapy	2.7 (0.8)	2.8 (0.9)	2.9 (0.9)	3.2* (0.9)	0.002

BMI, body mass index.

* 100 or more patients are significantly different from 10 to 25 patients.

† 100 or more patients are significantly different from 26 to 59 patients.

## Discussion

This study indicates that while PCPs share some beliefs about initiating insulin, there is a lack of consensus about other aspects of insulin therapy. Most shared beliefs fall into one of four categories: benefits of insulin therapy vs. risks, positive experiences of patients on insulin, fears or concerns of patients still on oral therapy, and the management of and training for insulin use.

The majority of PCPs agreed that the benefits of using insulin to prevent or delay complications outweighed the risks of hypoglycaemia and weight gain for most patients. However, there was less consensus when the patient was severely obese or elderly. For example, while the clear majority of PCPs agreed that the benefits of insulin outweighed the risks of hypoglycaemia for most patients, 44% agreed that the risk of hypoglycaemia made them reluctant to prescribe insulin to most patients who were ≥ 85 years old. The risk of hypoglycaemia is greater in elderly patients who have poor or erratic nutritional intake and/or comorbidities (12) and impaired recovery from hypoglycaemia (13,14). However, there is no evidence to suggest that the treatment goal for otherwise healthy elderly patients should differ from that for younger patients (HbA1c ≤ 7%), and the PCPs in this study agreed.

Most PCPs agreed that patients feel much better after they have begun insulin and that patients can manage the demands of insulin. Several studies have confirmed that patients, including elderly patients, experience reduction in fatigue and increased feelings of well-being when they begin insulin and that these improvements are sustained over time (15–18). Most PCPs also agreed that patients on insulin were satisfied with their insulin therapy. Studies show that patients on insulin, regardless of delivery mode (vial and syringe, pen or inhalation), have high levels of treatment satisfaction (19–21).

In a review of medication–adherence literature for patients with type 2 diabetes, Rubin (22) concluded that the adherence rate for oral antihyperglycaemic medication was approximately 65–85%, and insulin adherence may be slightly lower. In this study, nearly two-thirds of PCPs believed that their insulin-using patients were adherent, and only about a quarter of PCPs agreed that their patients on oral therapy would be less adherent to insulin therapy. Because the potential benefit patients receive from a treatment can be largely dependent on their adherence (22), further research is needed to determine whether PCP perceptions of patient adherence to insulin are accurate. Most PCPs agreed that patients on oral therapy are afraid of insulin injections and that this fear is a barrier to initiating insulin. PCPs were also largely in agreement that patients on oral therapy would be reluctant to initiate insulin and would have feelings of personal failure. These general patient concerns are well documented in the literature (3–5). Nearly all PCPs agreed that for most patients, education is the key to insulin initiation. However, Brunton et al. (23) pointed out that this education is usually given when diabetes has progressed to the point that insulin is the only alternative for glucose control. They further stressed the importance of educating the patient at diagnosis about the disease progression of diabetes and the inevitability of needing insulin to maintain good glycaemic control, rather than using insulin as a threat to motivate patients.

Although Riddle (6) identified the complexity of training patients in the proper use of insulin as a contributing factor to its under-use, more than half of the PCPs disagreed that training was too complicated for patients or that follow-up was too resource-intensive for their staff. However, there was no consensus that the time needed for training in the proper administration and usage of insulin was too much for their staff. This is not surprising as educational resources available to PCPs for insulin initiation vary widely.

Primary care physicians also clearly lacked consensus on whether patients on insulin performed self-monitoring of blood glucose (SMBG) sufficiently for appropriate insulin use. Appropriate SMBG frequency varies according to insulin regimen: three or four times daily is recommended for multiple injections, less frequent monitoring is needed for less intensive therapy (24). However, SMBG in patients with type 2 diabetes is often suboptimal (25). Reimbursement and resources for SMBG instruction may be highly variable, and SMBG adds to the patient's ‘hassle factor’. These issues may contribute to lack of PCP consensus about patient SMBG sufficiency, but patient fear of SMBG probably does not influence this PCP belief (26).

Primary care physicians exhibited a clear dichotomy concerning whether adherence to a diabetes regimen or following physician's recommendations would prevent patients with type 2 diabetes from requiring insulin. Disagreement with the first belief and agreement with the second raise the question of whether these PCPs respondents truly understood the progressive nature of diabetes. Because of the continuing decline in insulin secretion, within 6–10 years after diagnosis (sooner if the patient had type 2 diabetes for years prior to diagnosis) as many as 40–60% of patients with type 2 diabetes will need insulin to maintain glycaemic control (27,28), regardless of adherence to medication regimens and/or following physician recommendations.

The three items with unimodal response distribution were based on Riddle's (6) observations that many physicians worry that insulin therapy results in negative metabolic effects. The results of the United Kingdom Prospective Diabetes Study (29) and the Diabetes, Insulin-Glucose, And Myocardial Infarction (30) studies led Riddle to conclude that there is ‘compelling evidence’ that insulin treatment is not harmful with respect to cardiovascular disease and is most likely beneficial. A more recent retrospective observational study using a national health-claims database (31) reported the probability of a cardiovascular event to be 34% less for patients with type 2 diabetes on insulin than for those not on insulin. PCP beliefs about negative metabolic effects of insulin may be based on lack of knowledge and indicate a need for continuing medical education.

Two-thirds of the PCPs in this study agreed with the statement ‘…the initiation of insulin is one of the most difficult aspects of managing my patients with type 2 diabetes’. When associations between PCP characteristics and beliefs were examined, very little difference was seen in beliefs by gender or type of board certification. However, PCP attitudes varied by both years of practice and average number of patients with type 2 diabetes seen per week. PCPs with more years of practice had more positive attitudes about patients on insulin than PCPs with less experience, perhaps simply because of more long-term experience with such patients. PCPs with the least practice experience disagreed more strongly than more experienced PCPs that insulin has negative metabolic effects. The beliefs of the least experienced may reflect the fact that the ‘compelling evidence’ mentioned by Riddle (6) has been incorporated into medical student and resident education.

Primary care physicians who treated greater numbers of patients with type 2 diabetes per week appeared to be more risk-averse concerning insulin initiation compared with those who saw fewer patients, indicating that the diabetes care of many patients with type 2 diabetes is being managed by PCPs who have beliefs that suggest a reluctance to initiate insulin therapy in such patients.

A limitation of this study is that the data were self-reported and may differ from actual practice patterns of the study participants. However, other work has shown that physician attitudes are closely linked to behaviour (32), leading us to assume that many PCPs delay the prescribing of insulin to patients with type 2 diabetes. Another limitation is that, according to the AMA master file of 2000 (33), the percentage of female physicians in primary care (defined by AMA as general and family practice, internal medicine, obstetrics/gynaecology and paediatrics) in 2000 was approximately 34%. In our study, the percentage of female PCPs was 20%. Therefore, the attitudes of female PCPs may not have been sufficiently represented. Finally, another limitation is the survey administration: This study is generalisable to only those PCPs who have internet access and who would volunteer for such a study. Nevertheless, sampling was designed to ensure representation from all parts of the country and equal representation of family practitioners and internists.

## Conclusions

The clear majority of PCPs in this study indicated that their glycaemic control goal for patients of all ages with type 2 diabetes is HbA1c ≤ 7%. Given that insulin regimens are effective in reducing HbA1c levels, the findings suggest that some PCPs have beliefs about insulin that are barriers to attaining this glycaemic goal. Oral antihyperglycaemic therapies potentially delay but do not halt the progressive nature of diabetes; thus insulin therapy eventually will be needed by many patients with type 2 diabetes. The lack of consensus about insulin initiation identified in this study calls for continuing medical education programmes that increase PCP knowledge about diabetes and the physiological effects of insulin. These programmes should provide PCPs, especially those with large and challenging diabetes practices, with strategies and tools for successfully initiating insulin in patients with type 2 diabetes.
